# 1-(2*H*-1,3-Benzodioxol-5-yl)ethanone thio­semicarbazone

**DOI:** 10.1107/S1600536813008398

**Published:** 2013-04-05

**Authors:** Adriano Bof de Oliveira, Renan Lira de Farias, Christian Näther, Inke Jess, Leandro Bresolin

**Affiliations:** a Departamento de Química, Universidade Federal de Sergipe, Av. Marechal Rondon s/n, Campus, 49100-000 São Cristóvão-SE, Brazil; b Institut für Anorganische Chemie, Christian-Albrechts-Universität zu Kiel, Max-Eyth Strasse 2, D-24118 Kiel, Germany; c Escola de Química e Alimentos, Universidade Federal do Rio Grande, Av. Itália km 08, Campus Carreiros, 96201-900, Rio Grande-RS, Brazil

## Abstract

In the title compound, C_10_H_11_N_3_O_2_S, the 1,3-benzodioxole and hydrazinecarbothio­amide fragments are nearly planar [(mean deviations from planarity for non-H atoms of 0.0325 (12) Å and 0.0707 (10) Å, respectively] and subtend a dihedral angle of 29.06 (5)°. In the crystal, mol­ecules are linked by pairs of almost linear N—H⋯S hydrogen bonds, forming inversion dimers. These dimers are additionally connected by weaker and strongly bent N—H⋯S inter­actions into chains along [101]. There is one additional weak N—H⋯O contact which, if considered as an inter­action, leads to the formation of a three-dimensional network.

## Related literature

For the adapted synthesis of the title compound, see: de Oliveira *et al.* (2012 ▶). For the pharmacological activity of 3′,4′-(methyl­enedi­oxy)acetophenone thio­simecarbazone derivatives, see: Silva *et al.* (1998 ▶).
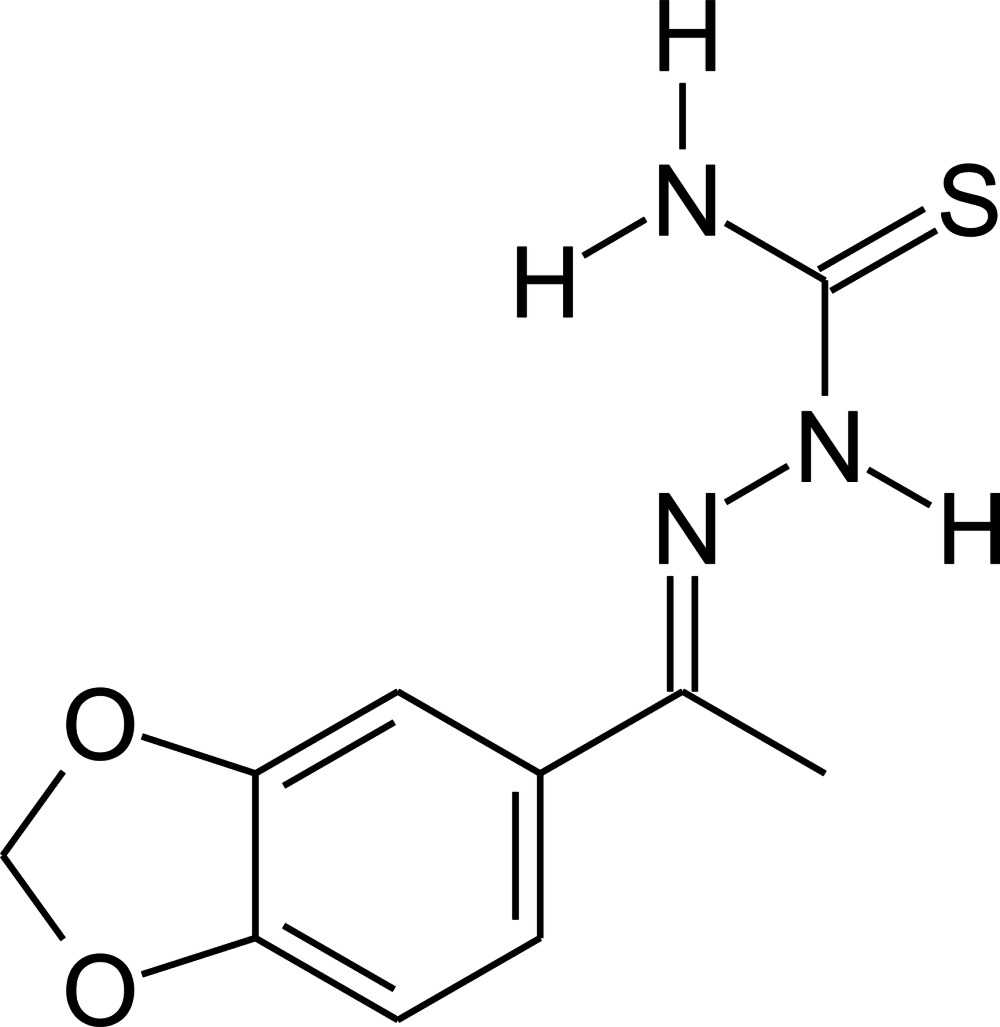


## Experimental

### 

#### Crystal data

C_10_H_11_N_3_O_2_S
*M* *_r_* = 237.28
Monoclinic, 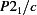

*a* = 6.1423 (12) Å
*b* = 26.065 (5) Å
*c* = 7.1289 (14) Å
β = 109.07 (3)°
*V* = 1078.7 (4) Å^3^
*Z* = 4
Mo *K*α radiation
μ = 0.29 mm^−1^
*T* = 200 K
0.3 × 0.2 × 0.2 mm

#### Data collection

Stoe IPDS-1 diffractometer
12310 measured reflections
2315 independent reflections
2018 reflections with *I* > 2σ(*I*)
*R*_int_ = 0.046

#### Refinement

*R*[*F* ^2^ > 2σ(*F* ^2^)] = 0.037
*wR*(*F* ^2^) = 0.107
*S* = 1.04
2315 reflections
147 parameters
H-atom parameters constrained
Δρ_max_ = 0.29 e Å^−3^
Δρ_min_ = −0.32 e Å^−3^

### 

Data collection: *X-AREA* (Stoe & Cie, 2008 ▶); cell refinement: *X-AREA*; data reduction: *X-RED32* (Stoe & Cie, 2008 ▶); program(s) used to solve structure: *SHELXS97* (Sheldrick, 2008 ▶); program(s) used to refine structure: *SHELXL97* (Sheldrick, 2008 ▶); molecular graphics: *DIAMOND* (Brandenburg, 2011 ▶); software used to prepare material for publication: *publCIF* (Westrip, 2010 ▶).

## Supplementary Material

Crystal structure: contains datablock(s) I, global. DOI: 10.1107/S1600536813008398/qk2055sup1.cif

Structure factors: contains datablock(s) I. DOI: 10.1107/S1600536813008398/qk2055Isup2.hkl

Supplementary material file. DOI: 10.1107/S1600536813008398/qk2055Isup3.cml

Additional supplementary materials:  crystallographic information; 3D view; checkCIF report

## Figures and Tables

**Table 1:** Hydrogen-bond geometry (Å, °)

*D*—H⋯*A*	*D*—H	H⋯*A*	*D*⋯*A*	*D*—H⋯*A*
N1—H1*N*1⋯O1^i^	0.88	2.66	3.2207 (17)	123
N1—H2*N*1⋯S1^ii^	0.88	2.53	3.4100 (18)	175
N2—H1*N*2⋯S1^iii^	0.88	2.84	3.5048 (15)	134

Symmetry codes: (i) 
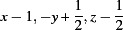
; (ii) 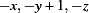; (iii) 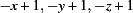.

**Fig. 1.**
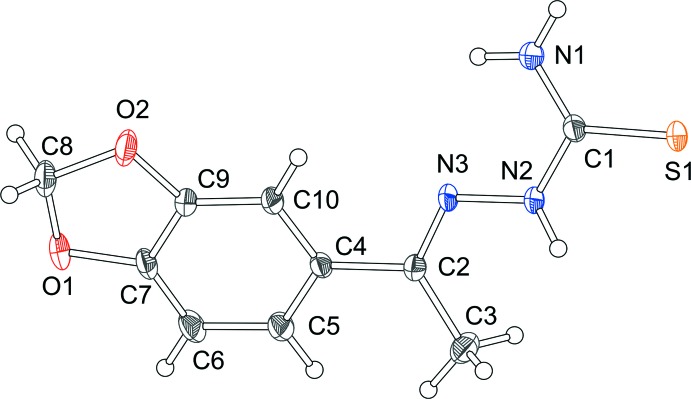
The molecular structure of the title compound with displacement ellipsoids drawn at the 40% probability level.

**Fig. 2.**
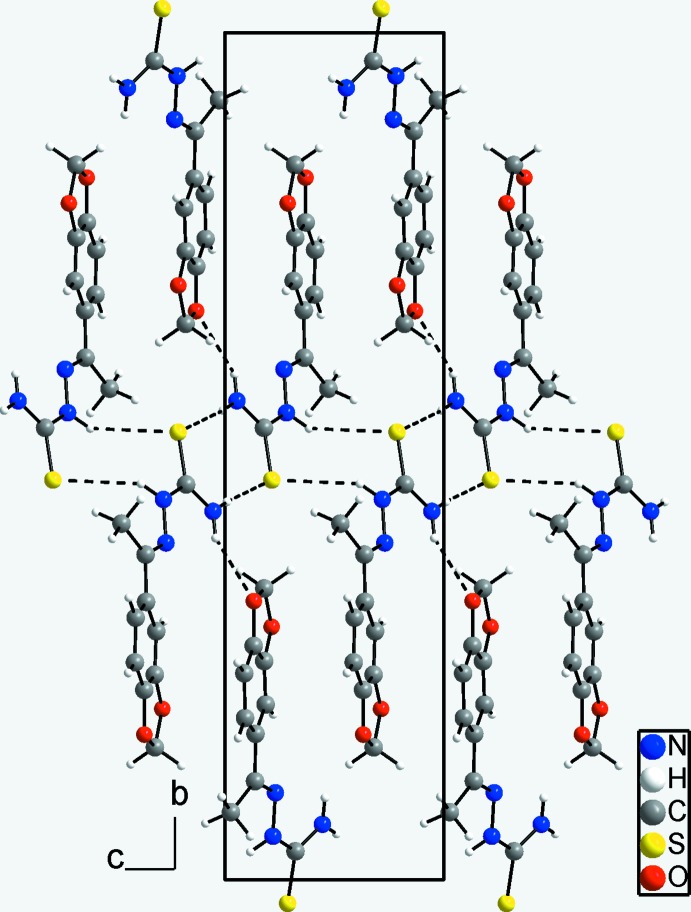
Crystal structure of the title compound in a view along the a axis with hydrogen bonds shown as dashed lines.

## supplementary crystallographic information

### Comment

Thiosemicarbazone derivatives have a wide range of pharmacological properties. For example, 3',4'-(methylenedioxy)acetophenone thiosemicarbazone derivatives show cytotoxic activity against KB cells (Silva *et al.*, 1998). As part of our study on the synthesis of thiosemicarbazone derivatives, we report herein the crystal structure of a new 3',4'-(methylenedioxy)acetophenone thiosemicarbazone derivative.

In the crystal structure of the title compound, C_10_H_11_N_3_O_2_S, the molecules are twisted and consists of two nearly planar parts, a benzo[1,3]dioxole fragment and a hydrazinecarbothioamide fragment (mean deviations from planarity for non-H atoms 0.0325 (12) Å and 0.0707 (10) Å, respectively), which subtend a dihedral angle of 29.06 (5)° (Fig. 1). The molecule shows an *E* conformation for the atoms about the N3—N2 and N2—C1 bonds, which is also observed in the crystal structures of other thiosemicarbazone derivatives (de Oliveira *et al.*, 2012).

In the crystal structure the molecules are linked by pairs of N—H···S hydrogen bonds into inversion related dimers (Fig. 2 and Table 1). These dimers are further connected by centrosymmetric pairs of weak N—H···S interactions into chains extending along [101] (Fig. 2). Finally, there is one N—H···O contact between that NH_2_ hydrogen atom which is not involved in N—H···S bonding and the dioxole oxygen O1 (Table 1). If this contact is considered as an interaction the chains are additionally linked into a three-dimensional network (Fig. 2).

In the crystal structure the benzo[1,3]dioxole fragments are oriented nearly perpendicular to [001] and are stacked above each other along this direction by the *c*-glide plane with a repetition period of *c*/2 = 3.5645 (7) Å implying π-π stacking.

### Experimental

All starting materials are commercially available and were used without further purification. The synthesis was adapted from a procedure reported previously (de Oliveira *et al.*, 2012). The hydrochloric acid catalyzed reaction of 3',4'-(methylenedioxy)acetophenone (10 mmol) and thiosemicarbazide (10 mmol) in a 3:1 mixture of ethanol and water (100 ml) was refluxed for 6 h. After cooling and filtering crystals suitable for X-ray diffraction were obtained.

Elemental analysis: Calc. 50.62% for C, 4.67% for H, 17.71% for N and 13.51% for S; found 50.72% for C, 4.66% for H, 17.70% for N and 13.47% for S. The melting point was determined by differential scanning calorimetry to 187° C and the enthalpy of fusion amount to 25.9 kJ/mol. After melting the compound decomposes.

### Refinement

All H atoms were located in a Fourier map. C-bonded H atoms were then geometrically placed (C—H = 0.95–0.99 Å) and refined as riding with *U*_iso_(H) = 1.2*U*_eq_(C(CH), C(CH_2_)) or 1.5*U*_eq_(C) and AFIX 136 for the methyl group. The N-bonded H atoms were only adjusted to N—H = 0.88 Å and were then refined as riding with *U*_iso_(H) = 1.2 *U*_eq_(N).

### Crystal data

**Table:**

C_10_H_11_N_3_O_2_S	*F*(000) = 496
*M**_r_* = 237.28	*D*_x_ = 1.461 Mg m^−^^3^
Monoclinic, *P*2_1_/*c*	Melting point: 460.2 K
Hall symbol: -P 2ybc	Mo *K*α radiation, λ = 0.71073 Å
*a* = 6.1423 (12) Å	Cell parameters from 8000 reflections
*b* = 26.065 (5) Å	θ = 3–27.9°
*c* = 7.1289 (14) Å	µ = 0.29 mm^−^^1^
β = 109.07 (3)°	*T* = 200 K
*V* = 1078.7 (4) Å^3^	Block, yellow
*Z* = 4	0.3 × 0.2 × 0.2 mm

### Data collection

**Table:**

Stoe IPDS-1 diffractometer	2018 reflections with *I* > 2σ(*I*)
Radiation source: fine-focus sealed tube	*R*_int_ = 0.046
Graphite monochromator	θ_max_ = 27.0°, θ_min_ = 3.1°
φ scans	*h* = −7→7
12310 measured reflections	*k* = −33→33
2315 independent reflections	*l* = −9→9

### Refinement

**Table:**

Refinement on *F*^2^	Secondary atom site location: difference Fourier map
Least-squares matrix: full	Hydrogen site location: inferred from neighbouring sites
*R*[*F*^2^ > 2σ(*F*^2^)] = 0.037	H-atom parameters constrained
*wR*(*F*^2^) = 0.107	*w* = 1/[σ^2^(*F*_o_^2^) + (0.0692*P*)^2^ + 0.2915*P*] where *P* = (*F*_o_^2^ + 2*F*_c_^2^)/3
*S* = 1.04	(Δ/σ)_max_ = 0.001
2315 reflections	Δρ_max_ = 0.29 e Å^−^^3^
147 parameters	Δρ_min_ = −0.32 e Å^−^^3^
0 restraints	Extinction correction: *SHELXL97* (Sheldrick, 2008), Fc^*^=kFc[1+0.001xFc^2^λ^3^/sin(2θ)]^-1/4^
Primary atom site location: structure-invariant direct methods	Extinction coefficient: 0.047 (8)

### Special details

**Table:**

Geometry. All e.s.d.'s (except the e.s.d. in the dihedral angle between two l.s. planes) are estimated using the full covariance matrix. The cell e.s.d.'s are taken into account individually in the estimation of e.s.d.'s in distances, angles and torsion angles; correlations between e.s.d.'s in cell parameters are only used when they are defined by crystal symmetry. An approximate (isotropic) treatment of cell e.s.d.'s is used for estimating e.s.d.'s involving l.s. planes.
Refinement. Refinement of *F*^2^ against ALL reflections. The weighted *R*-factor *wR* and goodness of fit *S* are based on *F*^2^, conventional *R*-factors *R* are based on *F*, with *F* set to zero for negative *F*^2^. The threshold expression of *F*^2^ > σ(*F*^2^) is used only for calculating *R*-factors(gt) *etc*. and is not relevant to the choice of reflections for refinement. *R*-factors based on *F*^2^ are statistically about twice as large as those based on *F*, and *R*- factors based on ALL data will be even larger.

### Fractional atomic coordinates and isotropic or equivalent isotropic displacement parameters (Å^2^)

**Table:**

	*x*	*y*	*z*	*U*_iso_*/*U*_eq_	
N1	0.2311 (2)	0.43475 (5)	0.0548 (2)	0.0326 (3)	
H1N1	0.2765	0.4032	0.0431	0.049*	
H2N1	0.0917	0.4461	−0.0094	0.049*	
C1	0.3775 (3)	0.46661 (5)	0.1778 (2)	0.0229 (3)	
S1	0.31425 (7)	0.528178 (13)	0.21371 (6)	0.03093 (17)	
N2	0.5891 (2)	0.44885 (4)	0.28111 (18)	0.0252 (3)	
H1N2	0.6685	0.4673	0.3840	0.030*	
N3	0.6401 (2)	0.39750 (4)	0.26964 (18)	0.0230 (3)	
C2	0.8498 (2)	0.38377 (5)	0.3628 (2)	0.0205 (3)	
C3	1.0366 (3)	0.42034 (6)	0.4726 (3)	0.0332 (4)	
H3A	1.0430	0.4489	0.3850	0.050*	
H3B	1.1849	0.4024	0.5151	0.050*	
H3C	1.0039	0.4336	0.5892	0.050*	
C4	0.8967 (2)	0.32787 (5)	0.3605 (2)	0.0201 (3)	
C5	1.1206 (3)	0.30922 (6)	0.4082 (2)	0.0276 (3)	
H5	1.2450	0.3329	0.4394	0.033*	
C6	1.1679 (3)	0.25646 (6)	0.4115 (3)	0.0320 (4)	
H6	1.3209	0.2440	0.4421	0.038*	
C7	0.9848 (3)	0.22397 (5)	0.3689 (2)	0.0250 (3)	
O1	0.9848 (2)	0.17122 (4)	0.36822 (19)	0.0362 (3)	
C8	0.7485 (3)	0.15623 (6)	0.3202 (3)	0.0329 (4)	
H8A	0.7026	0.1345	0.1997	0.039*	
H8B	0.7267	0.1362	0.4308	0.039*	
O2	0.6109 (2)	0.20154 (4)	0.2866 (2)	0.0409 (3)	
C9	0.7615 (3)	0.24196 (5)	0.3209 (2)	0.0241 (3)	
C10	0.7108 (3)	0.29321 (5)	0.3141 (2)	0.0242 (3)	
H10	0.5562	0.3049	0.2795	0.029*	

### Atomic displacement parameters (Å^2^)

**Table:**

	*U*^11^	*U*^22^	*U*^33^	*U*^12^	*U*^13^	*U*^23^
N1	0.0320 (7)	0.0199 (6)	0.0367 (8)	0.0067 (5)	−0.0014 (6)	−0.0073 (5)
C1	0.0269 (7)	0.0175 (6)	0.0227 (7)	0.0036 (5)	0.0060 (6)	0.0011 (5)
S1	0.0335 (3)	0.0145 (2)	0.0383 (3)	0.00688 (13)	0.00280 (18)	−0.00235 (13)
N2	0.0285 (7)	0.0133 (6)	0.0287 (7)	0.0050 (4)	0.0022 (5)	−0.0034 (4)
N3	0.0278 (6)	0.0128 (5)	0.0255 (6)	0.0044 (4)	0.0049 (5)	−0.0015 (4)
C2	0.0231 (7)	0.0162 (6)	0.0226 (7)	0.0011 (5)	0.0079 (6)	−0.0007 (5)
C3	0.0258 (8)	0.0250 (7)	0.0475 (10)	−0.0039 (6)	0.0101 (7)	−0.0084 (6)
C4	0.0220 (7)	0.0168 (6)	0.0204 (6)	0.0039 (5)	0.0056 (5)	0.0008 (5)
C5	0.0219 (7)	0.0230 (7)	0.0363 (8)	0.0034 (5)	0.0073 (6)	0.0012 (6)
C6	0.0251 (7)	0.0269 (8)	0.0429 (9)	0.0116 (6)	0.0095 (7)	0.0044 (6)
C7	0.0331 (8)	0.0172 (7)	0.0246 (7)	0.0099 (5)	0.0092 (6)	0.0030 (5)
O1	0.0434 (7)	0.0165 (5)	0.0473 (7)	0.0112 (4)	0.0129 (6)	0.0045 (4)
C8	0.0487 (10)	0.0156 (7)	0.0349 (9)	0.0047 (6)	0.0145 (8)	0.0013 (6)
O2	0.0333 (6)	0.0141 (5)	0.0703 (9)	−0.0002 (4)	0.0100 (6)	−0.0022 (5)
C9	0.0262 (7)	0.0161 (6)	0.0281 (7)	0.0012 (5)	0.0064 (6)	−0.0006 (5)
C10	0.0209 (7)	0.0170 (7)	0.0324 (8)	0.0043 (5)	0.0058 (6)	−0.0006 (5)

### Geometric parameters (Å, º)

**Table:**

N1—C1	1.324 (2)	C4—C10	1.408 (2)
N1—H1N1	0.8802	C5—C6	1.404 (2)
N1—H2N1	0.8800	C5—H5	0.9500
C1—N2	1.3494 (19)	C6—C7	1.361 (2)
C1—S1	1.6901 (14)	C6—H6	0.9500
N2—N3	1.3831 (16)	C7—O1	1.3749 (17)
N2—H1N2	0.8800	C7—C9	1.382 (2)
N3—C2	1.2921 (19)	O1—C8	1.432 (2)
C2—C4	1.4864 (17)	C8—O2	1.4261 (19)
C2—C3	1.500 (2)	C8—H8A	0.9900
C3—H3A	0.9800	C8—H8B	0.9900
C3—H3B	0.9800	O2—C9	1.3704 (18)
C3—H3C	0.9800	C9—C10	1.3690 (19)
C4—C5	1.392 (2)	C10—H10	0.9500
			
C1—N1—H1N1	118.7	C4—C5—H5	119.0
C1—N1—H2N1	117.9	C6—C5—H5	119.0
H1N1—N1—H2N1	123.4	C7—C6—C5	117.03 (13)
N1—C1—N2	118.10 (12)	C7—C6—H6	121.5
N1—C1—S1	123.79 (12)	C5—C6—H6	121.5
N2—C1—S1	118.11 (11)	C6—C7—O1	128.48 (14)
C1—N2—N3	119.75 (12)	C6—C7—C9	121.67 (13)
C1—N2—H1N2	116.1	O1—C7—C9	109.84 (13)
N3—N2—H1N2	120.3	C7—O1—C8	105.81 (11)
C2—N3—N2	116.40 (12)	O2—C8—O1	108.23 (12)
N3—C2—C4	115.46 (12)	O2—C8—H8A	110.1
N3—C2—C3	123.80 (13)	O1—C8—H8A	110.1
C4—C2—C3	120.72 (13)	O2—C8—H8B	110.1
C2—C3—H3A	109.5	O1—C8—H8B	110.1
C2—C3—H3B	109.5	H8A—C8—H8B	108.4
H3A—C3—H3B	109.5	C9—O2—C8	106.15 (13)
C2—C3—H3C	109.5	C10—C9—O2	127.67 (14)
H3A—C3—H3C	109.5	C10—C9—C7	122.40 (14)
H3B—C3—H3C	109.5	O2—C9—C7	109.93 (12)
C5—C4—C10	119.61 (13)	C9—C10—C4	117.38 (13)
C5—C4—C2	121.26 (13)	C9—C10—H10	121.3
C10—C4—C2	119.12 (12)	C4—C10—H10	121.3
C4—C5—C6	121.91 (14)		
			
N1—C1—N2—N3	−5.7 (2)	C6—C7—O1—C8	178.09 (16)
S1—C1—N2—N3	174.06 (10)	C9—C7—O1—C8	−0.96 (17)
C1—N2—N3—C2	175.99 (13)	C7—O1—C8—O2	1.91 (17)
N2—N3—C2—C4	175.80 (11)	O1—C8—O2—C9	−2.14 (17)
N2—N3—C2—C3	−2.5 (2)	C8—O2—C9—C10	−177.54 (15)
N3—C2—C4—C5	163.15 (13)	C8—O2—C9—C7	1.57 (18)
C3—C2—C4—C5	−18.5 (2)	C6—C7—C9—C10	−0.3 (2)
N3—C2—C4—C10	−18.52 (19)	O1—C7—C9—C10	178.77 (13)
C3—C2—C4—C10	159.82 (14)	C6—C7—C9—O2	−179.51 (15)
C10—C4—C5—C6	0.0 (2)	O1—C7—C9—O2	−0.39 (18)
C2—C4—C5—C6	178.38 (14)	O2—C9—C10—C4	178.34 (15)
C4—C5—C6—C7	−1.0 (2)	C7—C9—C10—C4	−0.7 (2)
C5—C6—C7—O1	−177.77 (15)	C5—C4—C10—C9	0.8 (2)
C5—C6—C7—C9	1.2 (2)	C2—C4—C10—C9	−177.57 (13)

### Hydrogen-bond geometry (Å, º)

**Table:**

*D*—H···*A*	*D*—H	H···*A*	*D*···*A*	*D*—H···*A*
N1—H1*N*1···O1^i^	0.88	2.66	3.2207 (17)	123
N1—H2*N*1···S1^ii^	0.88	2.53	3.4100 (18)	175
N2—H1*N*2···S1^iii^	0.88	2.84	3.5048 (15)	134

Symmetry codes: (i) *x*−1, −*y*+1/2, *z*−1/2; (ii) −*x*, −*y*+1, −*z*; (iii) −*x*+1, −*y*+1, −*z*+1.
